# Supplementary material for: Statistical inference with a manifold-constrained RNA velocity model uncovers cell cycle speed modulations
Source: Nat Methods. 2024 Oct 31;21(12):2271–86. doi: 10.1038/s41592-024-02471-8 (PMC11621032; doi:10.1038/s41592-024-02471-8)
Supplement: Supplementary file 1 — Reporting Summary [file 41592_2024_2471_MOESM1_ESM.pdf]

nature portfolio

Corresponding author(s): Gioele La Manno
Felix Naef

Last updated by author(s): Jul 25, 2024

Reporting Summary

Nature Portfolio wishes to improve the reproducibility of the work that we publish. This form provides structure for consistency and transparency in reporting. For further information on Nature Portfolio policies, see our Editorial Policies and the Editorial Policy Checklist.

Statistics

For all statistical analyses, confirm that the following items are present in the figure legend, table legend, main text, or Methods section.

n/a	Confirmed	
☐	☒	The exact sample size (*n*) for each experimental group/condition, given as a discrete number and unit of measurement
☐	☒	A statement on whether measurements were taken from distinct samples or whether the same sample was measured repeatedly
☐	☒	The statistical test(s) used AND whether they are one- or two-sided<br>*Only common tests should be described solely by name; describe more complex techniques in the Methods section.*
☒	☐	A description of all covariates tested
☒	☐	A description of any assumptions or corrections, such as tests of normality and adjustment for multiple comparisons
☐	☒	A full description of the statistical parameters including central tendency (e.g. means) or other basic estimates (e.g. regression coefficient) AND variation (e.g. standard deviation) or associated estimates of uncertainty (e.g. confidence intervals)
☐	☒	For null hypothesis testing, the test statistic (e.g. *F*, *t*, *r*) with confidence intervals, effect sizes, degrees of freedom and *P* value noted<br>*Give P values as exact values whenever suitable.*
☐	☒	For Bayesian analysis, information on the choice of priors and Markov chain Monte Carlo settings
☒	☐	For hierarchical and complex designs, identification of the appropriate level for tests and full reporting of outcomes
☐	☒	Estimates of effect sizes (e.g. Cohen's *d*, Pearson's *r*), indicating how they were calculated

Our web collection on statistics for biologists contains articles on many of the points above.

Software and code

Policy information about availability of computer code

Data collection	VeloCycle is implemented in Python and available as an open-source package on GitHub at https://github.com/lamanno-lab/velocycle. VeloCycle can be installed from PyPi using the command pip install velocycle or via direct installation from the GitHub page using the command pip install git+https://github.com/lamanno-epfl/velocycle.git@main. Source code, installation instructions, tutorials, and a requirements.txt file containing all necessary package version dependencies are also available on GitHub. Tutorials can be found at the link: https://github.com/lamanno-epfl/velocycle/tree/main/tutorials
Data analysis	Analyses performed in this study were completed using VeloCycle v0.1.0.5. Additional code and notebooks to reproduce the results of this study are available on Zenodo with the following registered DOI: 10.5281/zenodo.12517650.<br><br>Python version 3.8 or newer is required. Analyses were performed using the following version of major packages: numpy (v1.24.4), pandas (v2.0.3), scanpy (v1.9.6), matplotlib (v3.7.4), pyro-ppl (v1.8.6), pyro-api (v.0.1.2), scikit-learn (v1.3.2), torch (v2.0.1), and pycircstat (v0.0.2).<br><br>To process raw fastq files into spliced and unspliced count matrices, we either used processed files available from previous studies or ran CellRanger (v6.0.2) and velocyto (v0.17.17). For phase estimation with DeepCycle, we used the latest version of the software, installed via GitHub from commit a33701a.

For manuscripts utilizing custom algorithms or software that are central to the research but not yet described in published literature, software must be made available to editors and reviewers. We strongly encourage code deposition in a community repository (e.g. GitHub). See the Nature Portfolio guidelines for submitting code & software for further information.

Data

Policy information about availability of data

All manuscripts must include a data availability statement. This statement should provide the following information, where applicable:

- Accession codes, unique identifiers, or web links for publicly available datasets
- A description of any restrictions on data availability
- For clinical datasets or third party data, please ensure that the statement adheres to our policy

The raw and processed scRNA-seq data in the RPE1 cell line that was newly generated for this study are available at GEO accession number GSE250148. All other scRNA-seq data used in this study were collected from previously published works (see publication for references) and relied on the cell type annotations made by the original authors.

Jupyter notebooks and other affiliated files to reproduce the results shown in this study are provided on Zenodo at the following DOI: 10.5281/zenodo.12517650. Processed versions of all published data (including spliced-unspliced counts matrices), simulated scRNA-seq datasets, processed scRNA-seq metadata for the new RPE1 samples, cell tracking data from live-image microscopy and cumulative EdU staining experiments are also on Zenodo.

Human research participants

Policy information about studies involving human research participants and Sex and Gender in Research.

Reporting on sex and gender	n/a
Population characteristics	n/a
Recruitment	n/a
Ethics oversight	n/a

Note that full information on the approval of the study protocol must also be provided in the manuscript.

Field-specific reporting

Please select the one below that is the best fit for your research. If you are not sure, read the appropriate sections before making your selection.

☒ Life sciences ☐ Behavioural & social sciences ☐ Ecological, evolutionary & environmental sciences

For a reference copy of the document with all sections, see nature.com/documents/nr-reporting-summary-flat.pdf

Life sciences study design

All studies must disclose on these points even when the disclosure is negative.

Sample size	No sample size calculation was performed, the study is about development and validation of a method.
Data exclusions	For scRNA-seq samples containing a mixture of cell types (i.e., human fibroblasts, developing mouse brain), cycling cells were isolated using the categorical cell type annotations provided by the authors of the studies in which the datasets were originally published. For the Perturb-seq dataset, all cells belonging to a knockdown condition that was represented by a total of at least 75 cells was retained. For all newly-generated scRNA-seq datasets, bad quality cells were filtered based on low UMIs and doubled were filtered based on high UMIs using standard practices of the community; genes were generally filtered based on an average spliced expression > 0.3 and an average unspliced expression > 0.1. More information on dataset specific filtering criteria are available in the Methods section and in the Jupyter notebooks used to perform the analysis, which are available at the Zenodo page.
Replication	For cell tracking experiments with RPE1 cells, between 20-25 cells were tracked from 15 different fields of view by three different individuals (A.R.L., A.H., A.V.) for a total of 337 cells used to estimate a ground truth cell cycle period.<br><br>For cumulative EdU and p21 experiments, 3 different experimental replicates (with 10, 10, and 9 time points each) were used to estimate the %p21 positive cells and the overall cell cycle time.
Randomization	For most analyses that were dataset-specific, randomization was not relevant to the analyses performed. For cell tracking experiments, three different individuals manually tracked between 20-25 randomly selected cells from 5 randomly chosen (but non-overlapping) fields of view.
Blinding	Blinding is not relevant to the experimental study presented in this work.

Reporting for specific materials, systems and methods

We require information from authors about some types of materials, experimental systems and methods used in many studies. Here, indicate whether each material, system or method listed is relevant to your study. If you are not sure if a list item applies to your research, read the appropriate section before selecting a response.

Materials & experimental systems

n/a	Involved in the study
☒	☐ Antibodies
☐	☒ Eukaryotic cell lines
☒	☐ Palaeontology and archaeology
☒	☐ Animals and other organisms
☒	☐ Clinical data
☒	☐ Dual use research of concern

Methods

n/a	Involved in the study
☒	☐ ChIP-seq
☒	☐ Flow cytometry
☒	☐ MRI-based neuroimaging

Eukaryotic cell lines

Policy information about cell lines and Sex and Gender in Research

Cell line source(s)	FUCCI-RPE1 cells (Battich et al. Science 2020) were obtained from the Tanenbaum Lab (Hubrecht Institute) via Oncode. To generate these cells, hTERT RPE-1 cells (ATCC; CRL-4000) were transduced with lentivirus expressing mkO2-hCdh1 (30/120) (FUCCI-G1) and mAG-hGem (1/110) (FUCCI-G2) (Sakaue-Sawano et al., 2008).
Authentication	None of the cell lines were authenticated by genome sequencing prior to experiments. However, RPE1 cells were maintained in culture at least for two passages prior to experiments to ensure culture stability.
Mycoplasma contamination	RPE1 cells were confirmed to be free of mycoplasma before proceeding with experiments.
Commonly misidentified lines (See ICLAC register)	No commonly misidentified cell lines were used in this study.
